# Supplementary material for: Genome-Wide Characterization of B-Box Gene Family in Salvia miltiorrhiza
Source: Int J Mol Sci. 2023 Jan 21;24(3):2146. doi: 10.3390/ijms24032146 (PMC9916448; doi:10.3390/ijms24032146)

Figure S1: Results of signal peptides analysis of *Salvia miltiorrhiza* BBX gene family.

SignalP-5.0 prediction (Eukarya): SmBBX1_

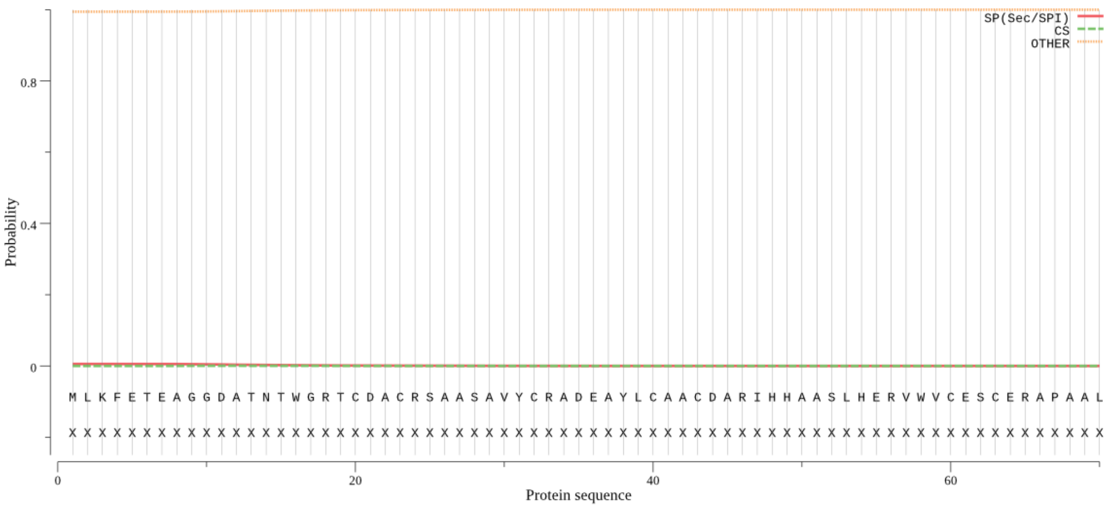

SignalP-5.0 prediction (Eukarya): SmBBX2_

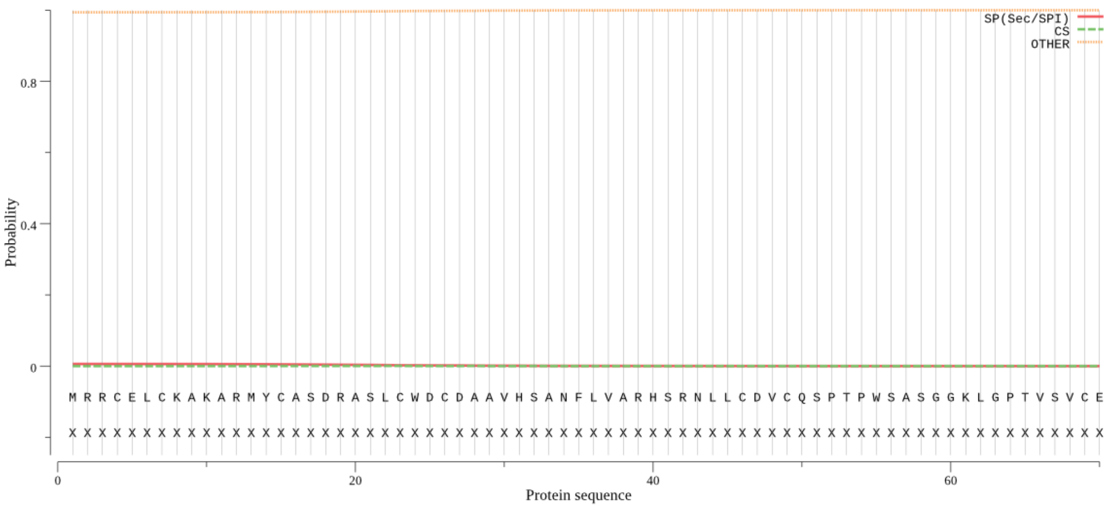

SignalP-5.0 prediction (Eukarya): SmBBX3_

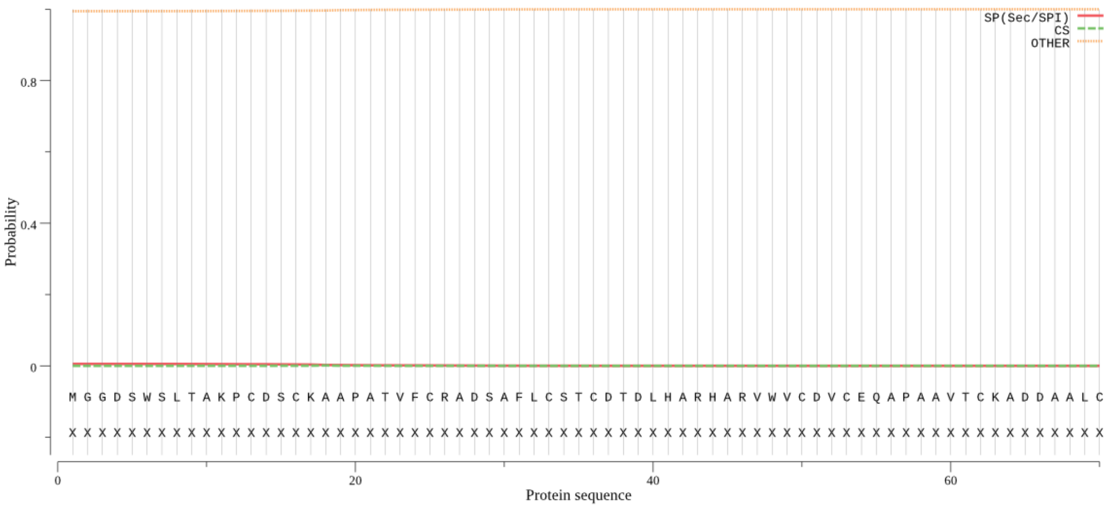

SignalP-5.0 prediction (Eukarya): SmBBX4_

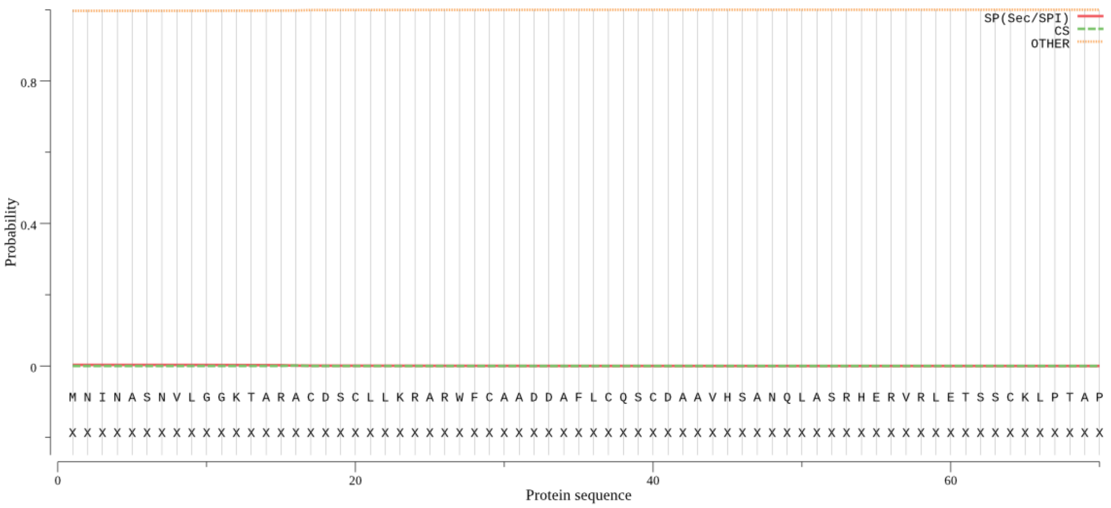

SignalP-5.0 prediction (Eukarya): SmBBX5_

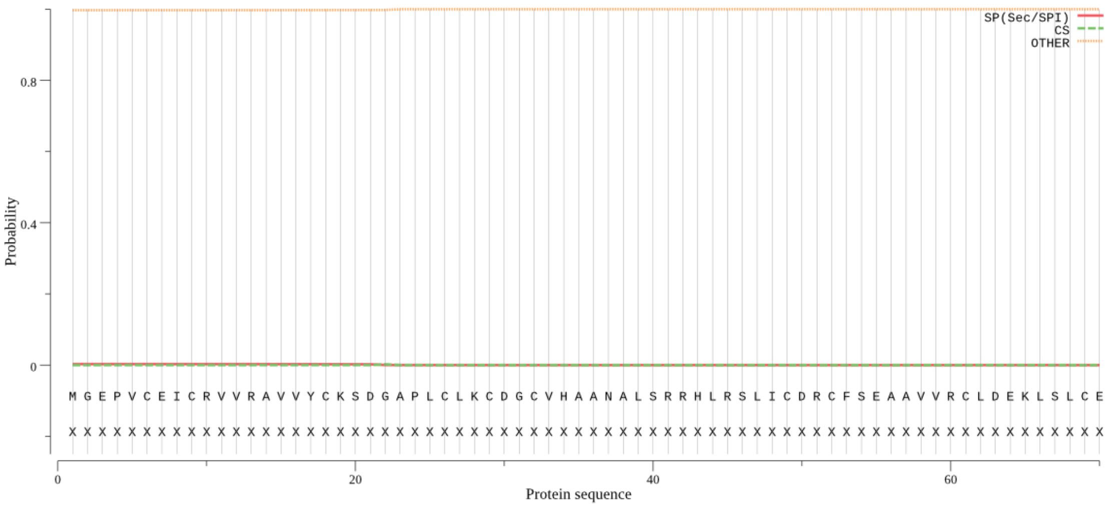

SignalP-5.0 prediction (Eukarya): SmBBX6_

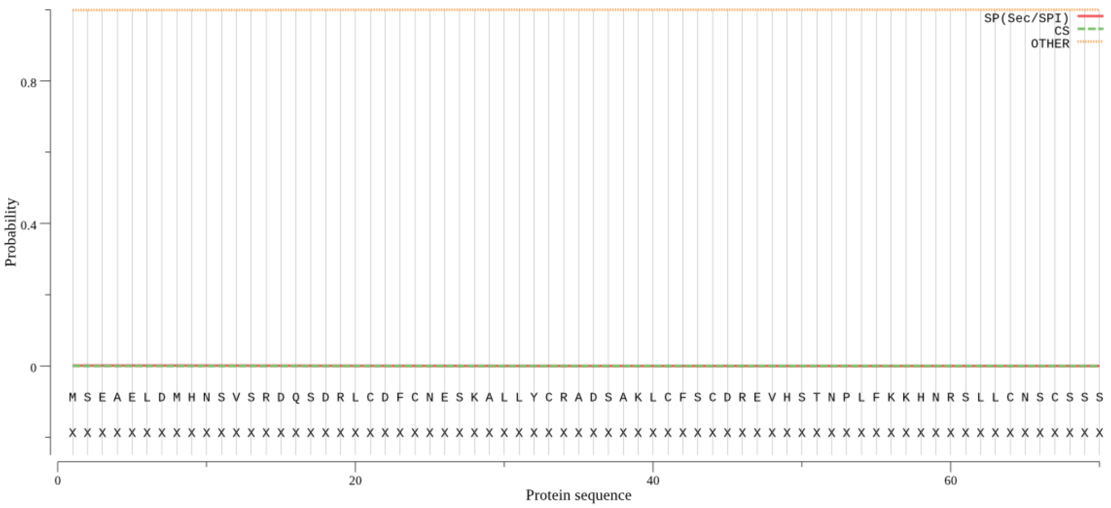

SignalP-5.0 prediction (Eukarya): SmBBX7_

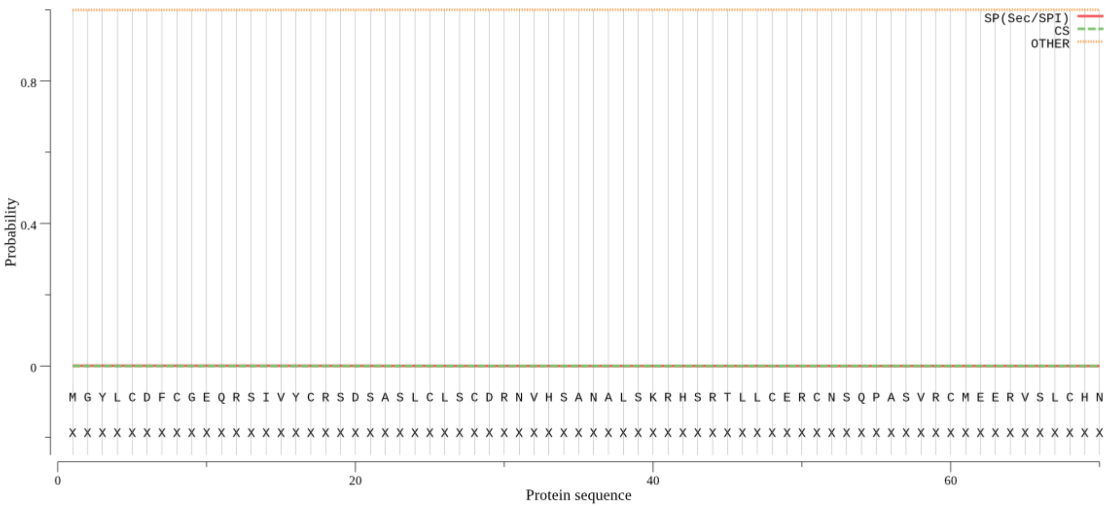

SignalP-5.0 prediction (Eukarya): SmBBX8_

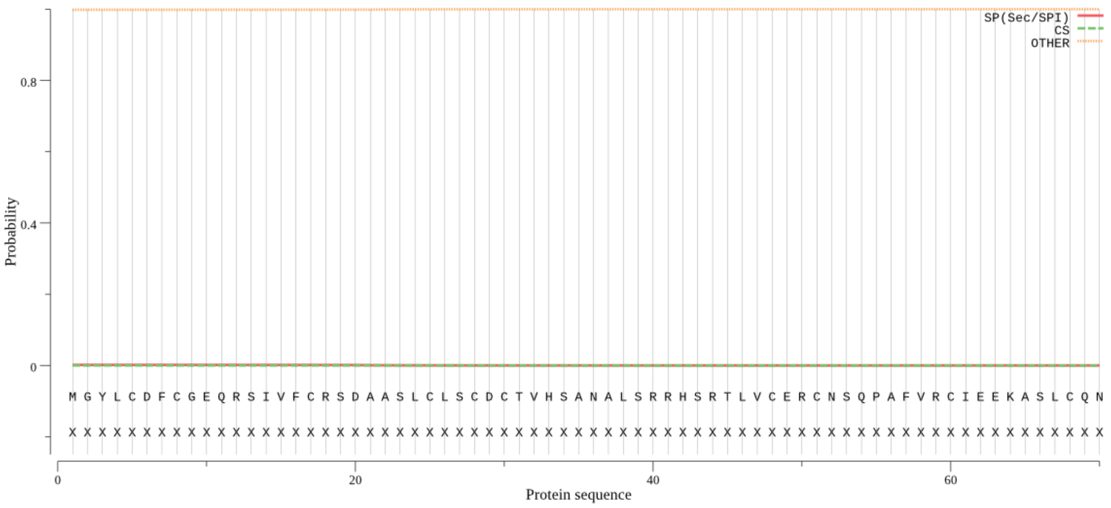

SignalP-5.0 prediction (Eukarya): SmBBX9_

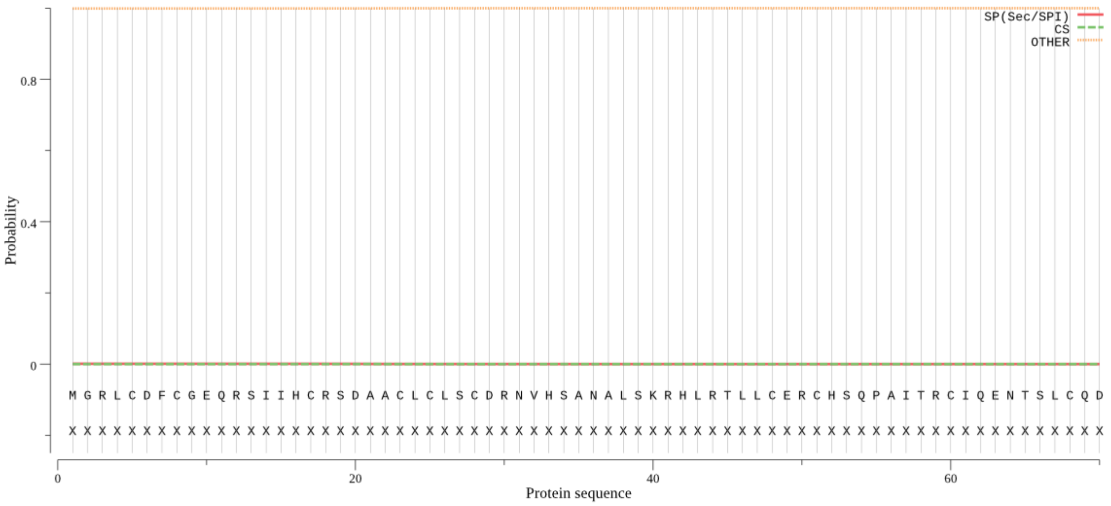

SignalP-5.0 prediction (Eukarya): SmBBX10_

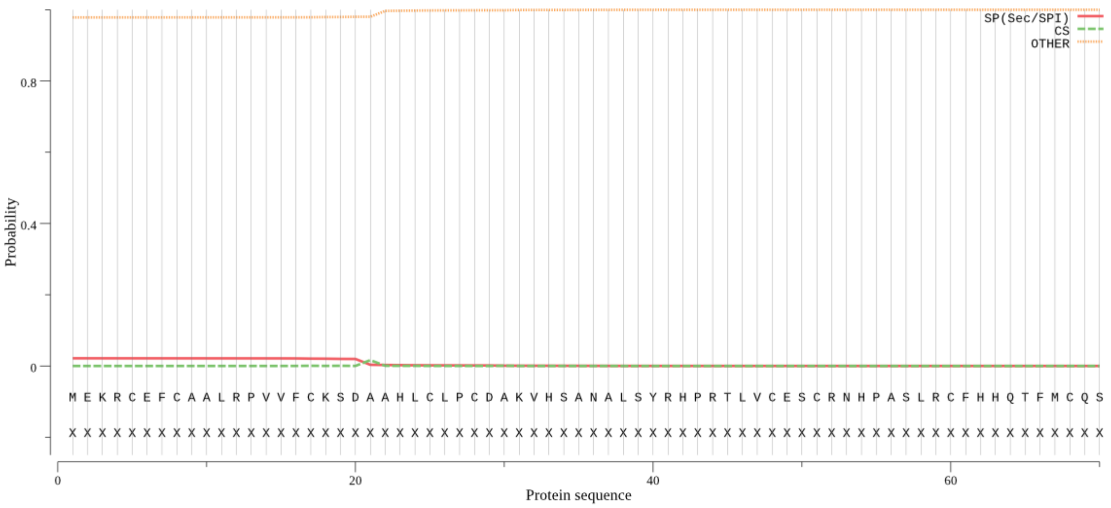

SignalP-5.0 prediction (Eukarya): SmBBX11_

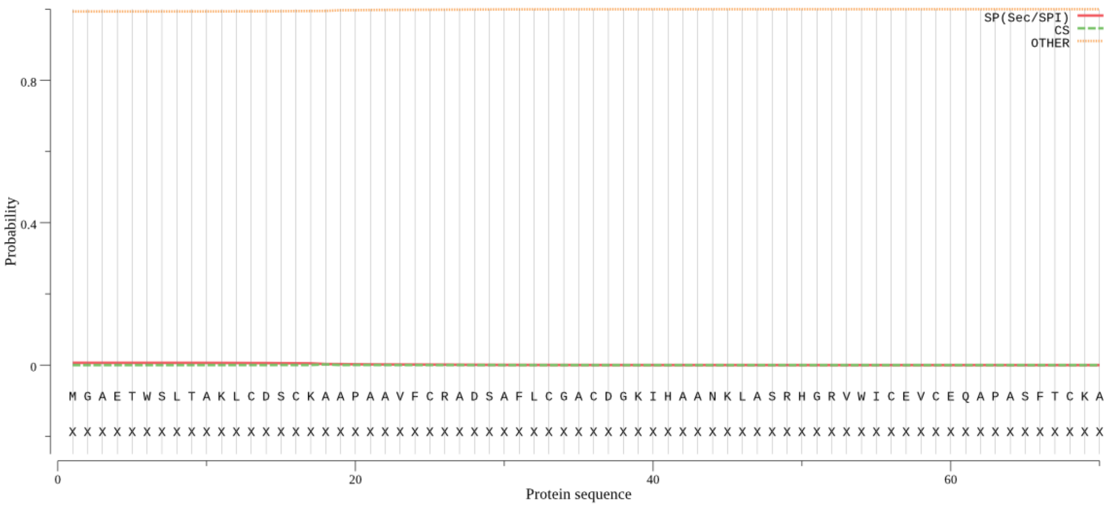

SignalP-5.0 prediction (Eukarya): SmBBX12_

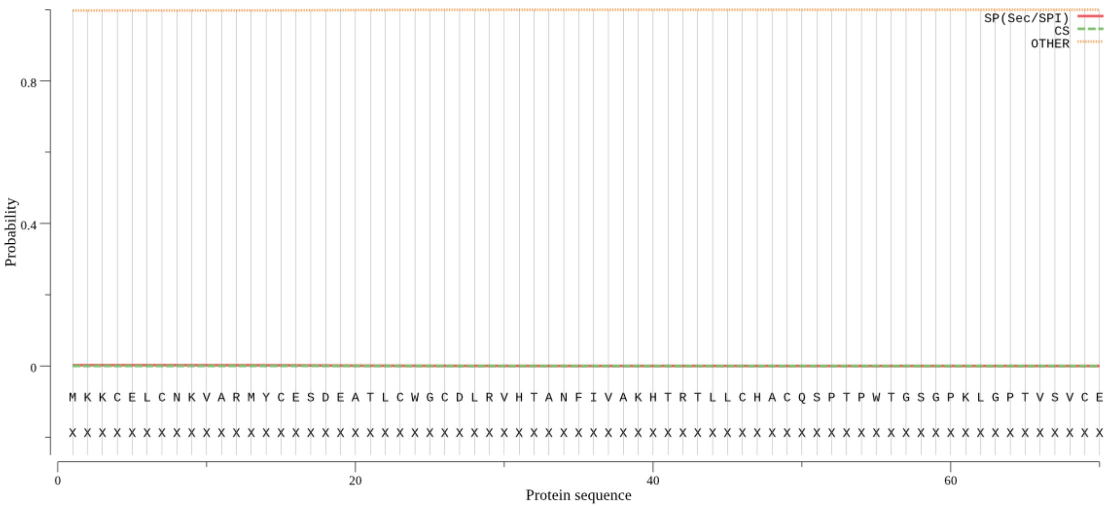

SignalP-5.0 prediction (Eukarya): SmBBX13_

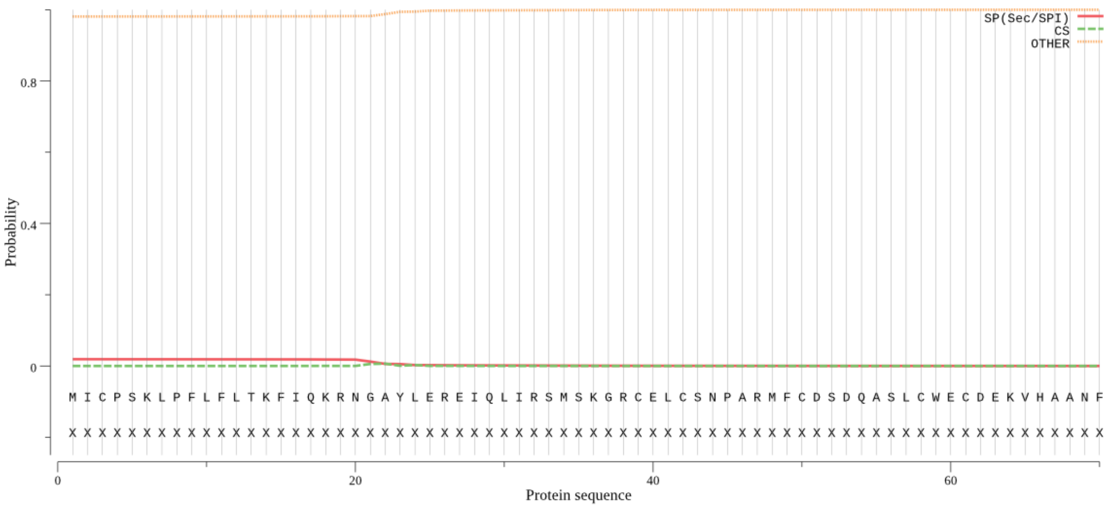

SignalP-5.0 prediction (Eukarya): SmBBX14

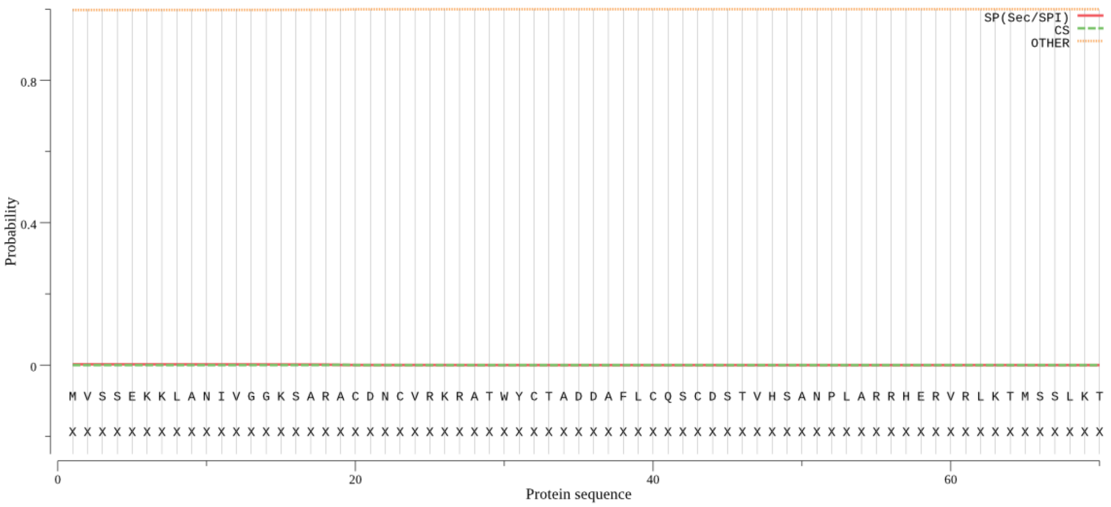

SignalP-5.0 prediction (Eukarya): SmBBX15_

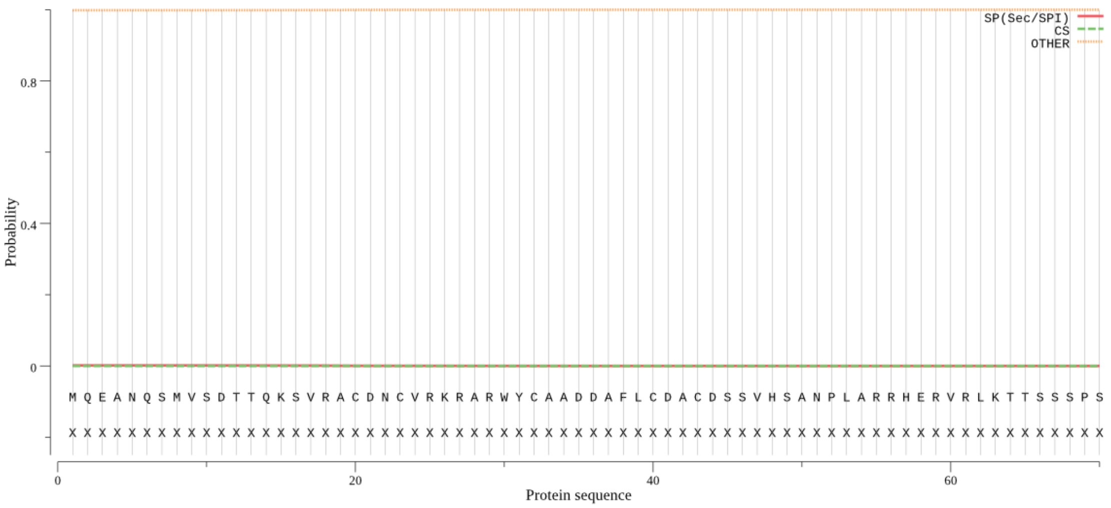

SignalP-5.0 prediction (Eukarya): SmBBX16_

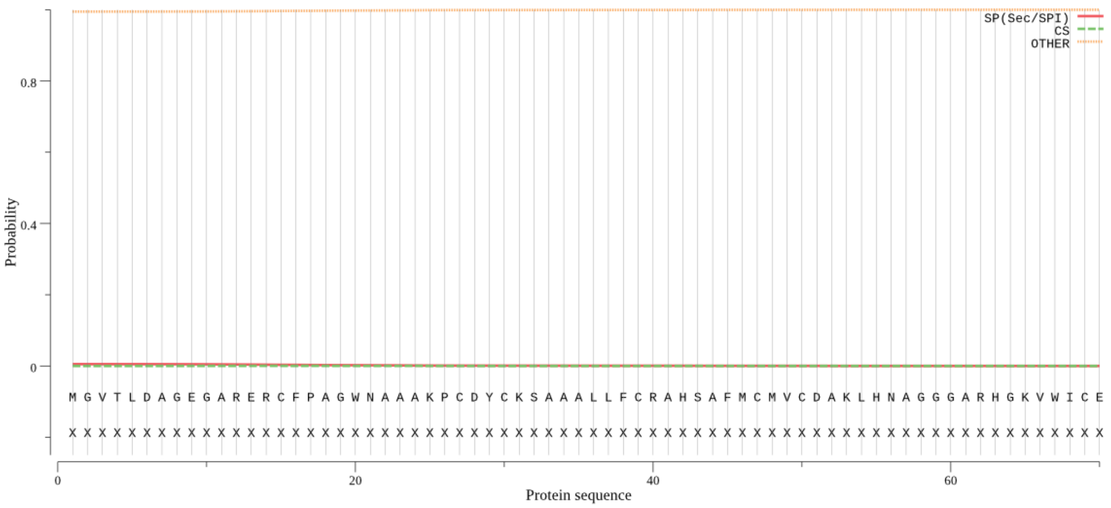

SignalP-5.0 prediction (Eukarya): SmBBX17

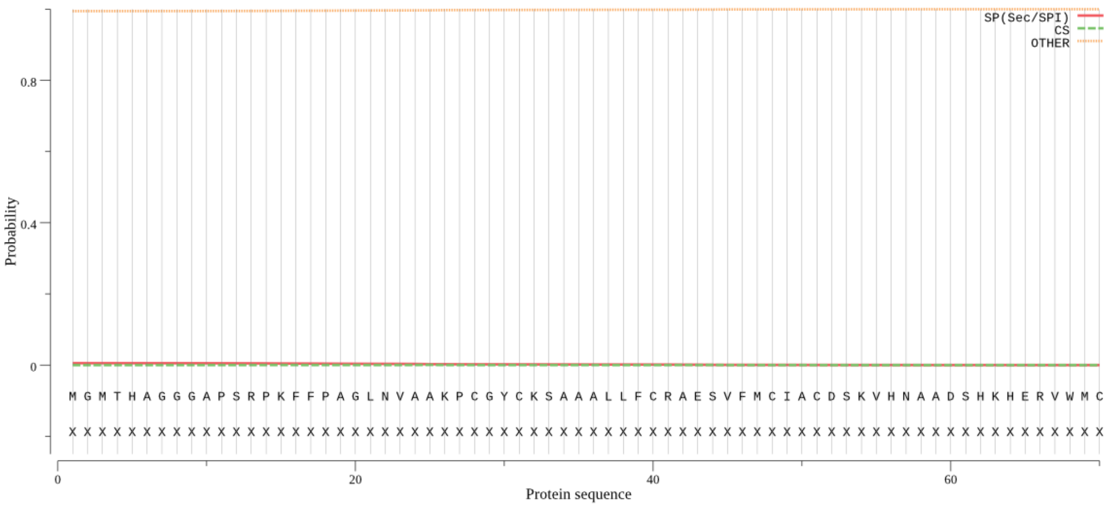

SignalP-5.0 prediction (Eukarya): SmBBX18_

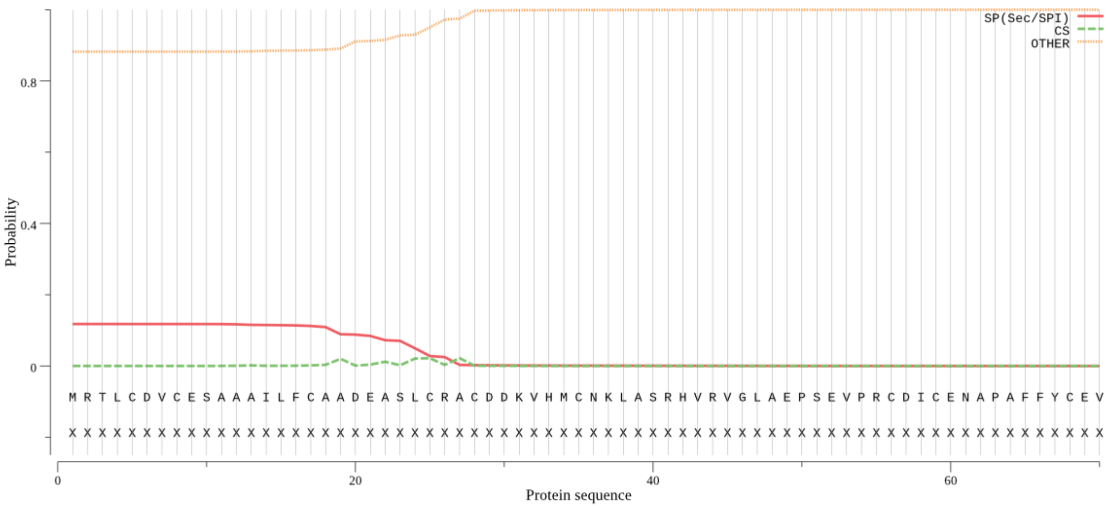

SignalP-5.0 prediction (Eukarya): SmBBX19_

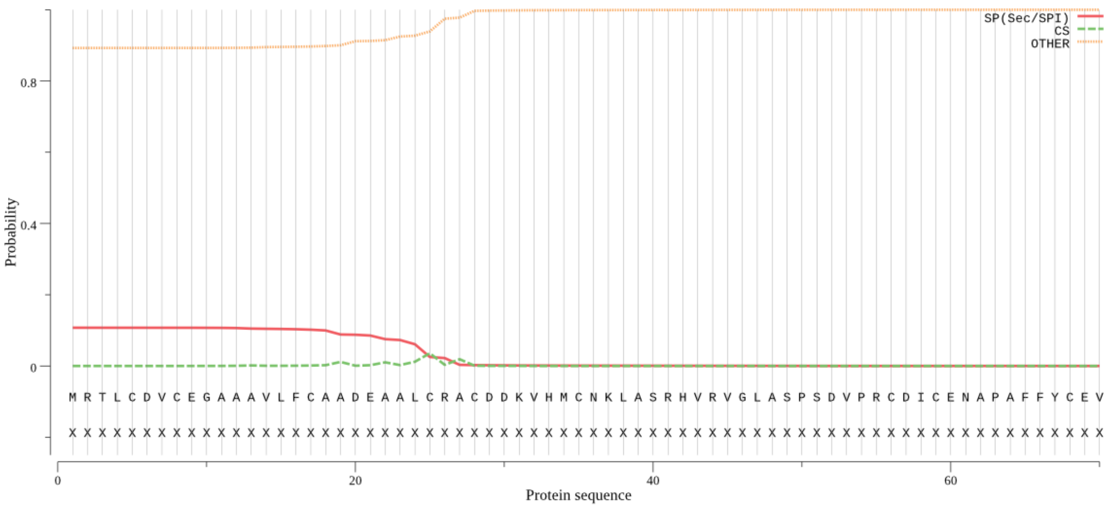

SignalP-5.0 prediction (Eukarya): SmBBX20

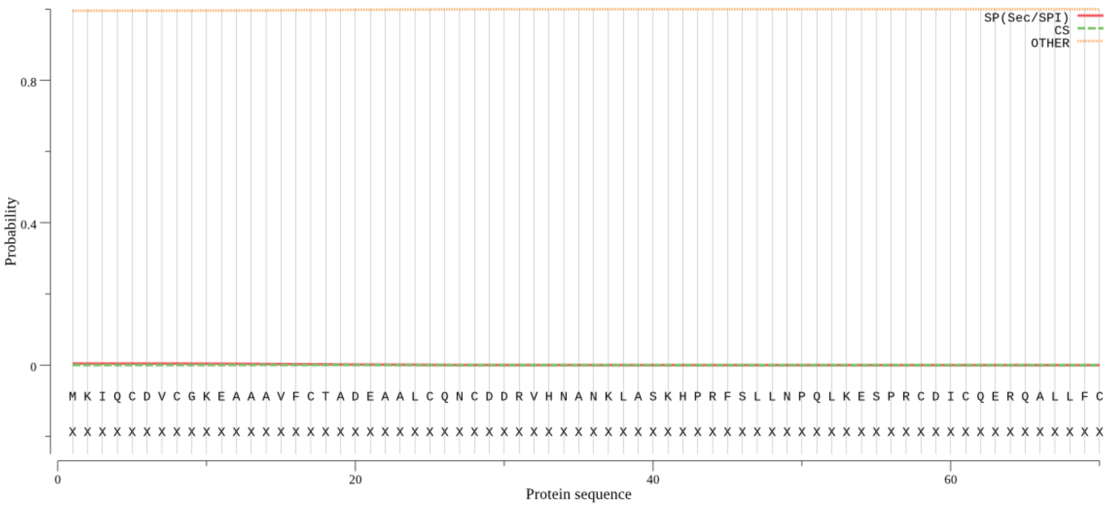

SignalP-5.0 prediction (Eukarya): SmBBX21_

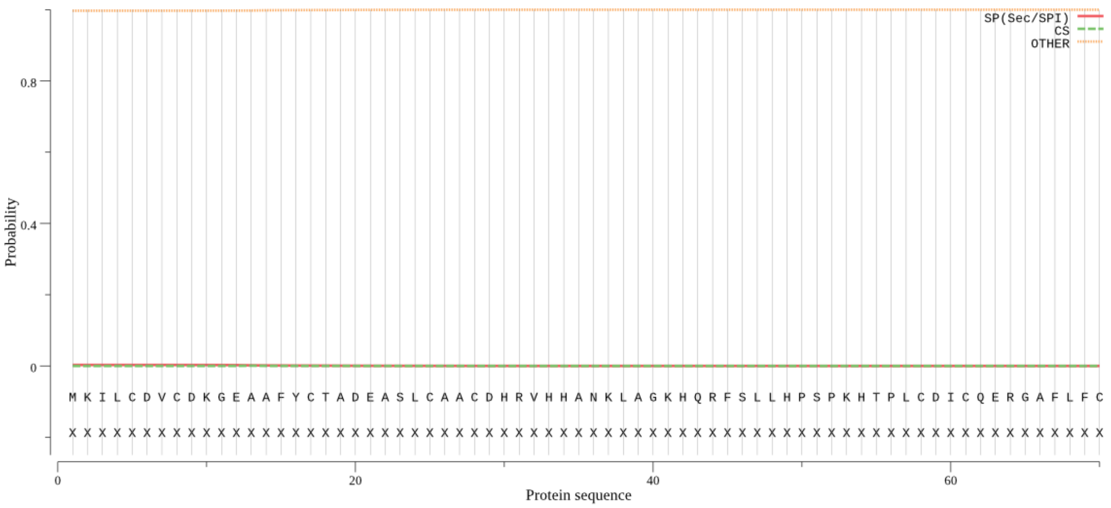

SignalP-5.0 prediction (Eukarya): SmBBX22

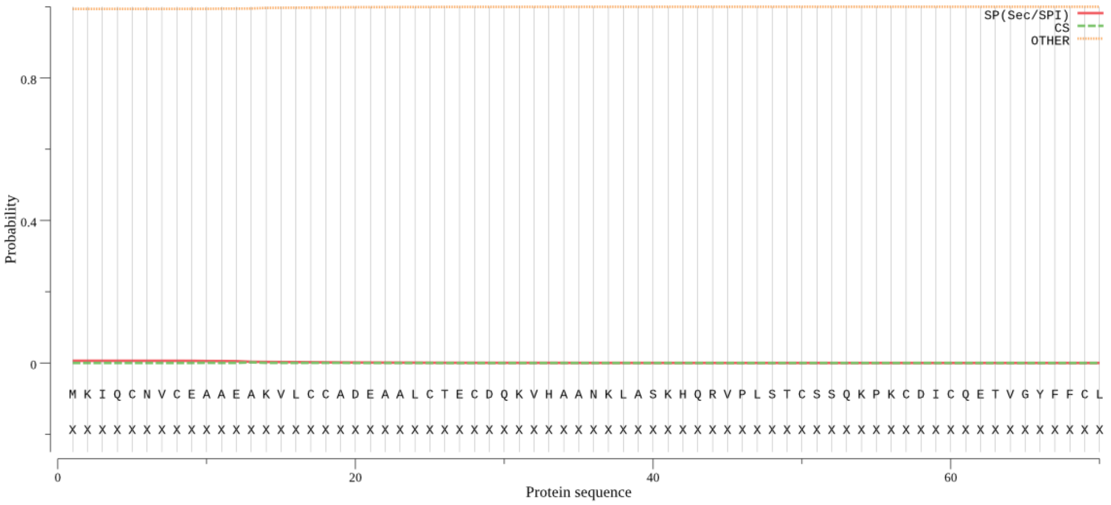

SignalP-5.0 prediction (Eukarya): SmBBX23_

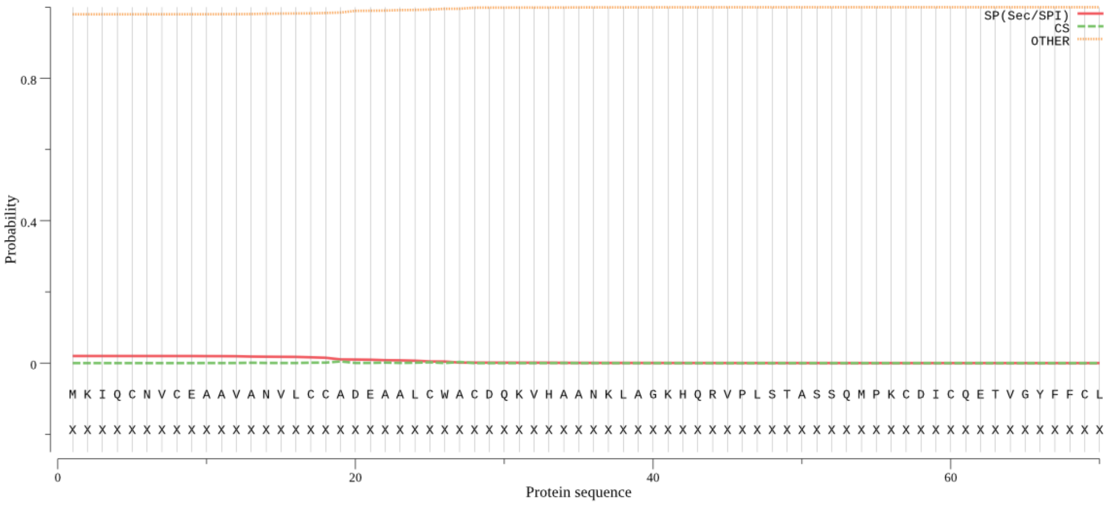

SignalP-5.0 prediction (Eukarya): SmBBX24_

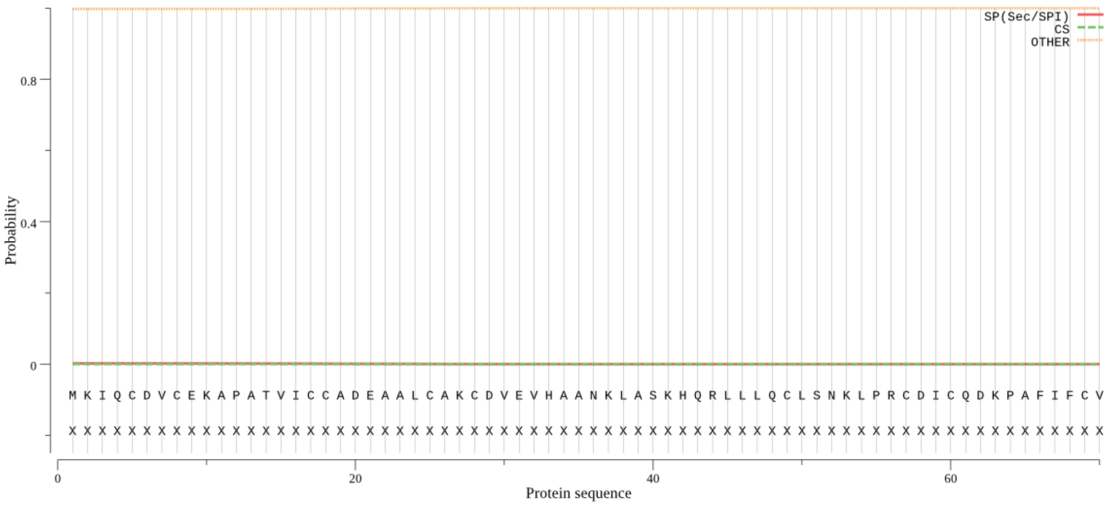

SignalP-5.0 prediction (Eukarya): SmBBX25_

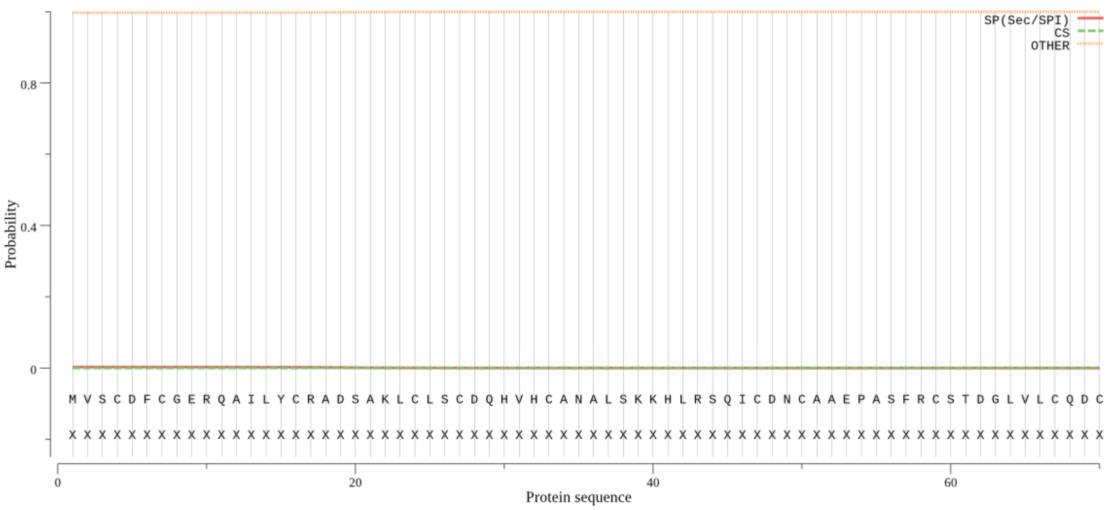

SignalP-5.0 prediction (Eukarya): SmBBX26_

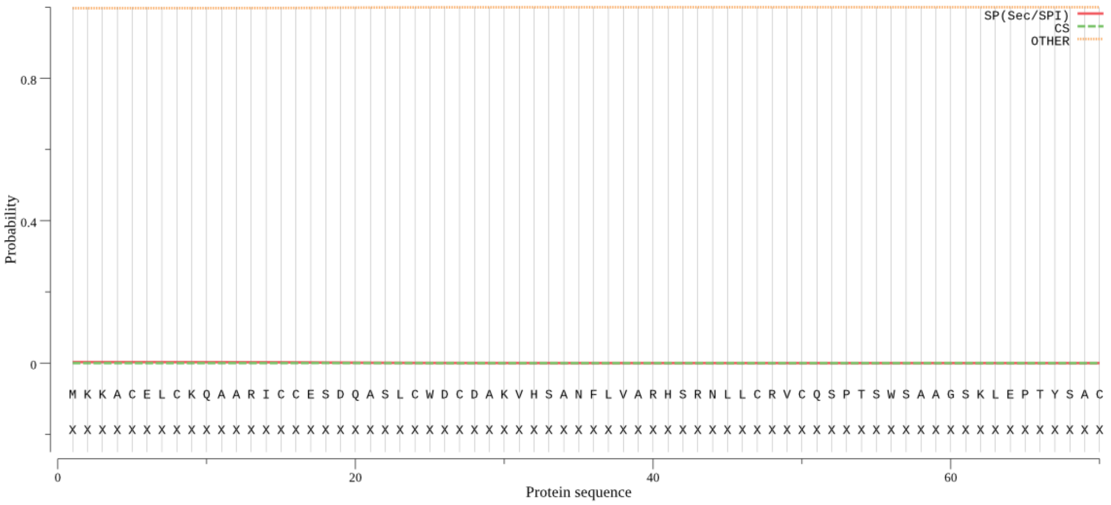

SignalP-5.0 prediction (Eukarya): SmBBX27_

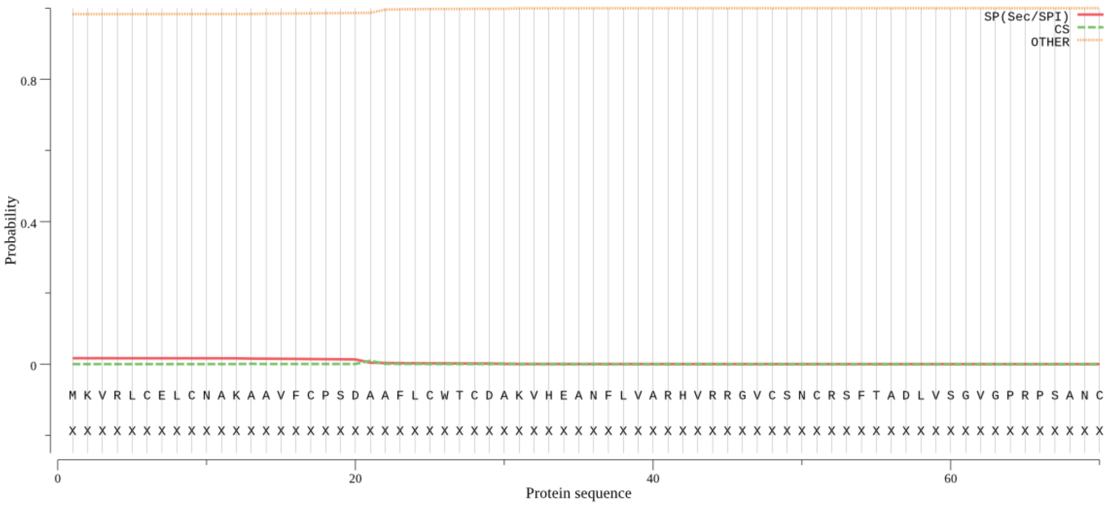

Supplement: Supplementary file 1 [file ijms-24-02146-s001.zip › Supplementary Figure S1.pdf]
