# Supplementary material for: Genome-Wide Characterization of B-Box Gene Family in Salvia miltiorrhiza
Source: Int J Mol Sci. 2023 Jan 21;24(3):2146. doi: 10.3390/ijms24032146 (PMC9916448; doi:10.3390/ijms24032146)
Supplement: Supplementary file 1 [file ijms-24-02146-s001.zip › Supplementary Figure S2.pdf]

**Figure S2:** Results of TMHMM transmembrane structure analysis of *Salvia miltiorrhiza* BBX gene family.

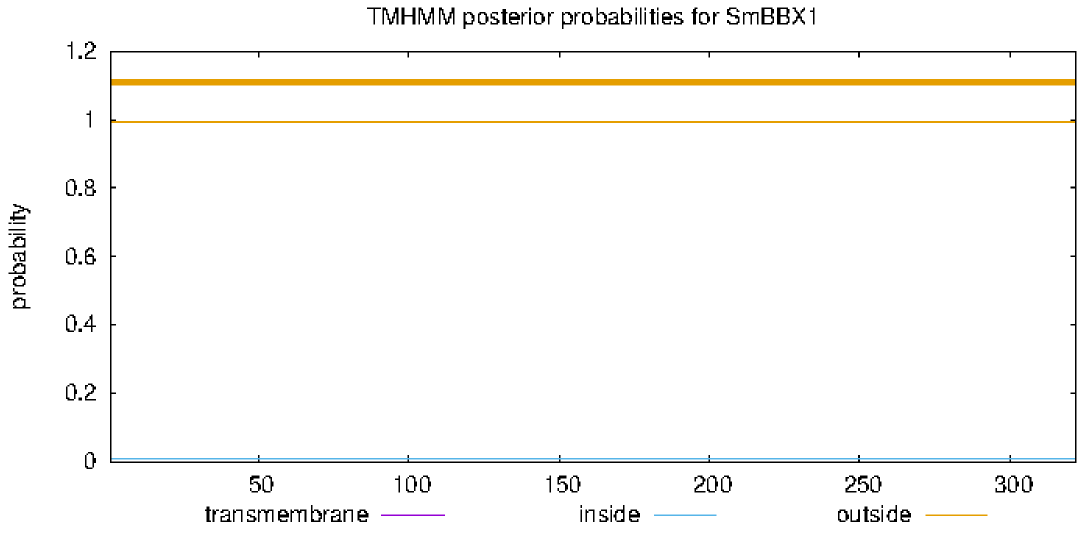


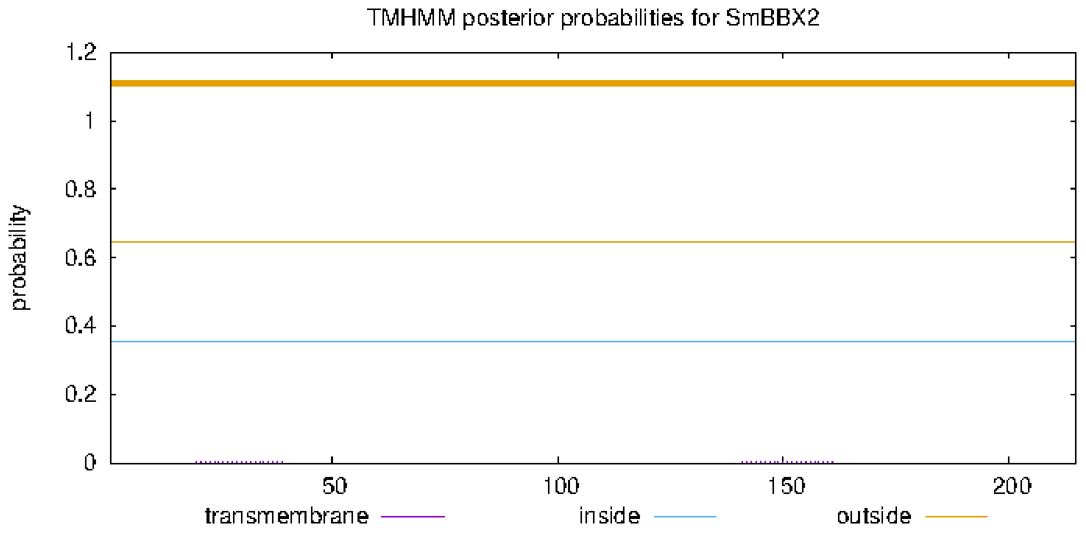


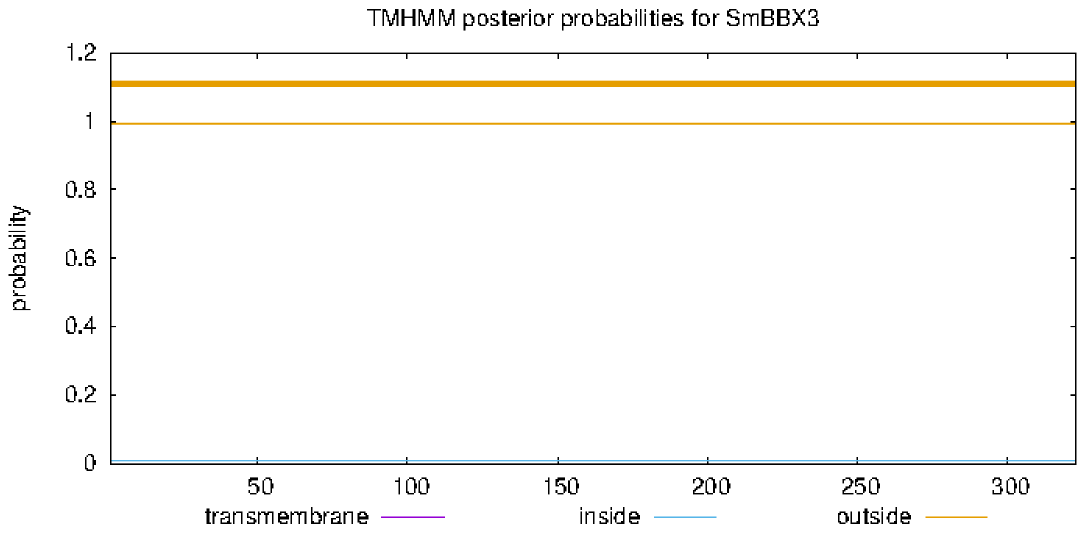

TMHMM posterior probabilities for SmBBX4

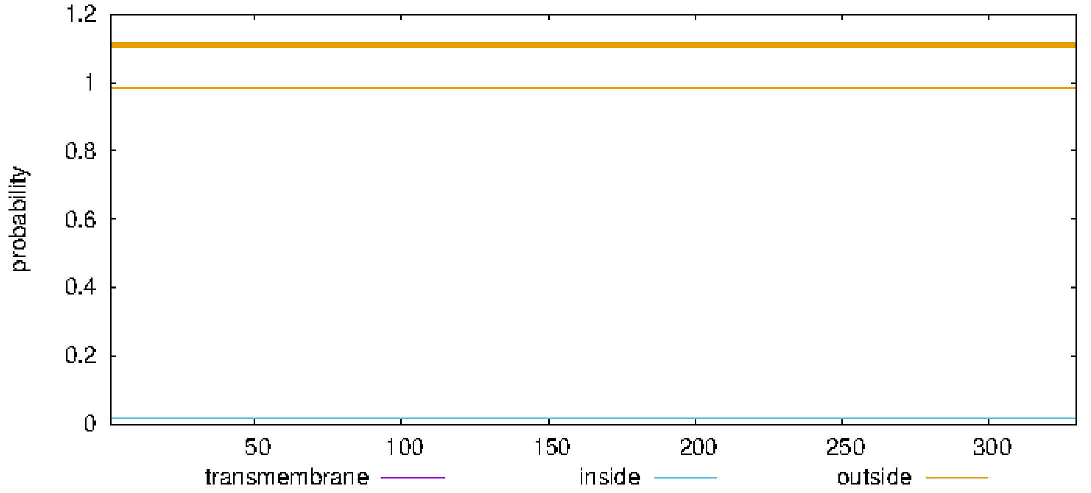


TMHMM posterior probabilities for SmBBX5

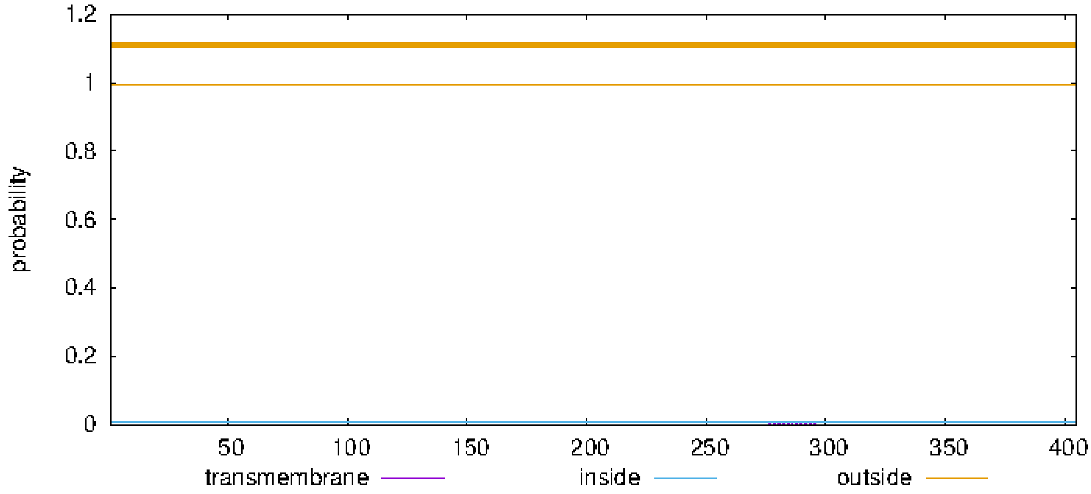


TMHMM posterior probabilities for SmBBX6

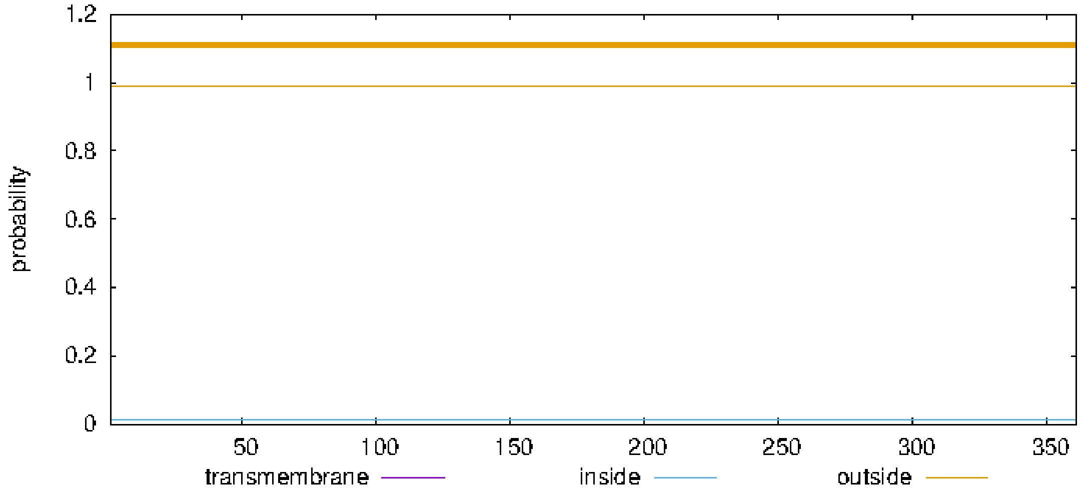

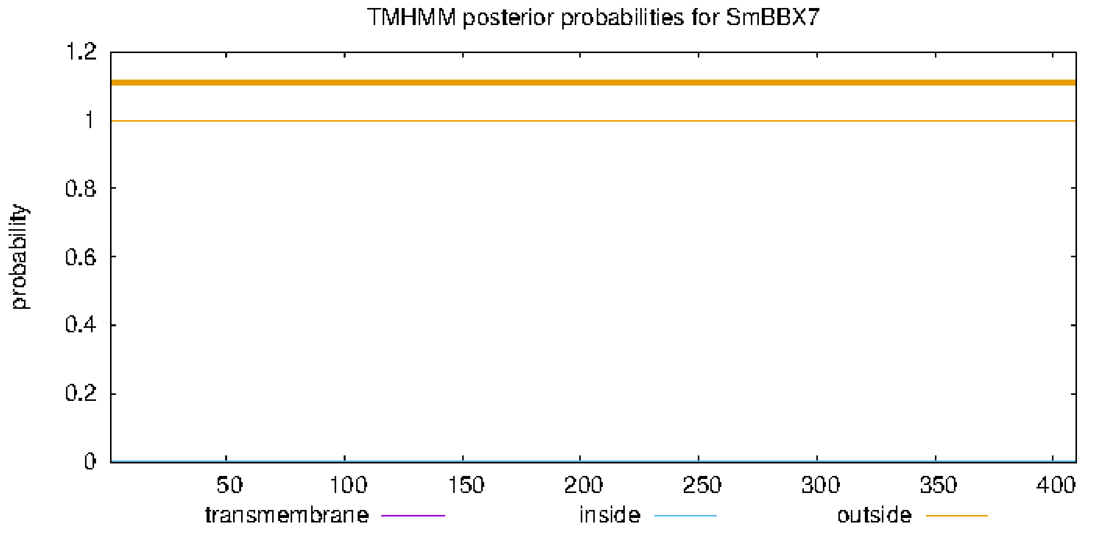
TMHMM posterior probabilities for SmBBX7
probability
1.2
1
0.8
0.6
0.4
0.2
0
50
100
150
200
250
300
350
400
transmembrane
inside
outside

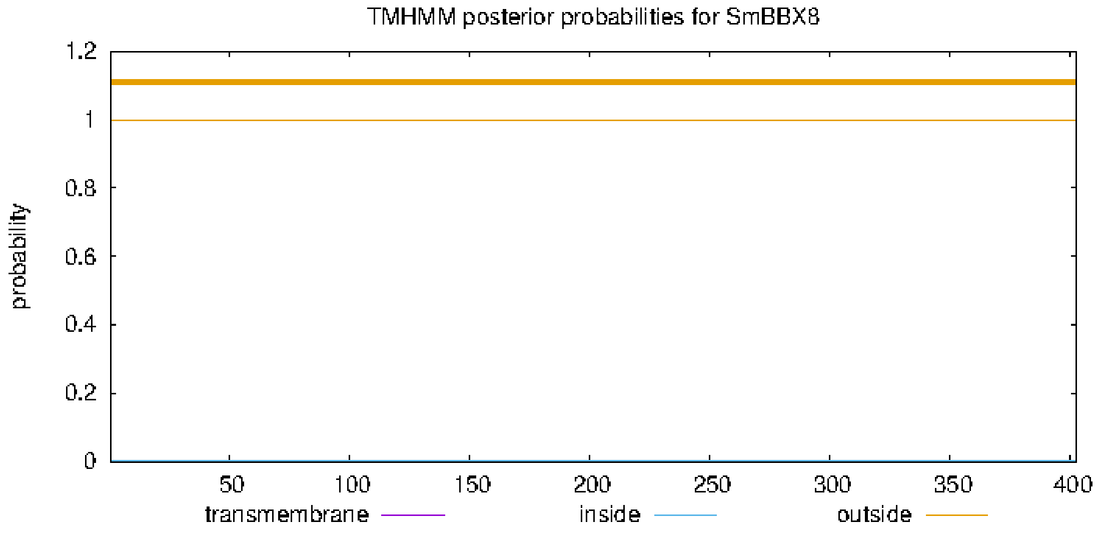
TMHMM posterior probabilities for SmBBX8
probability
1.2
1
0.8
0.6
0.4
0.2
0
50
100
150
200
250
300
350
400
transmembrane
inside
outside

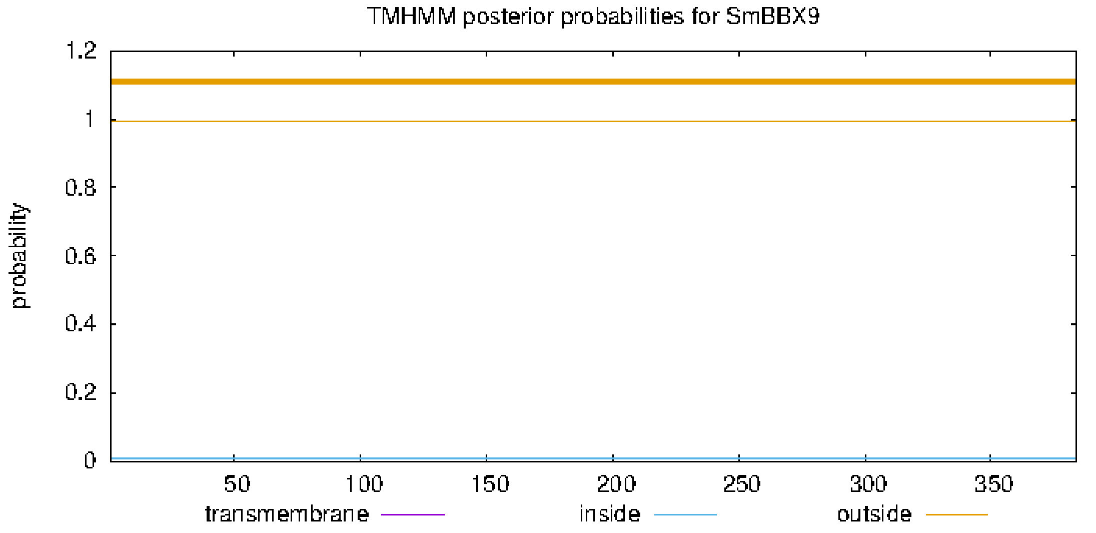
TMHMM posterior probabilities for SmBBX9
probability
1.2
1
0.8
0.6
0.4
0.2
0
50
100
150
200
250
300
350
transmembrane
inside
outside

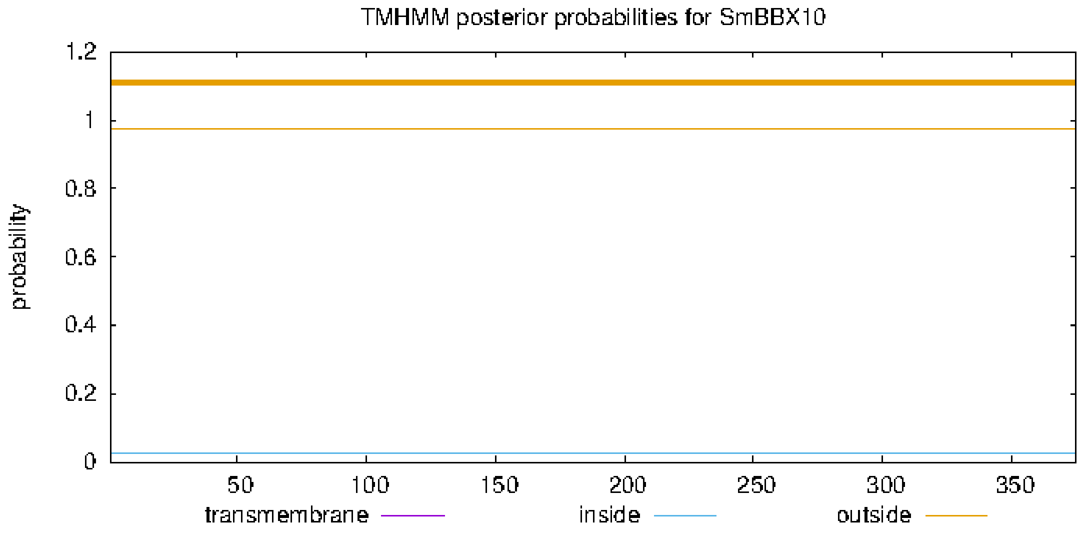
TMHMM posterior probabilities for SmBBX10
probability
1.2
1
0.8
0.6
0.4
0.2
0
50
100
150
200
250
300
350
transmembrane
inside
outside

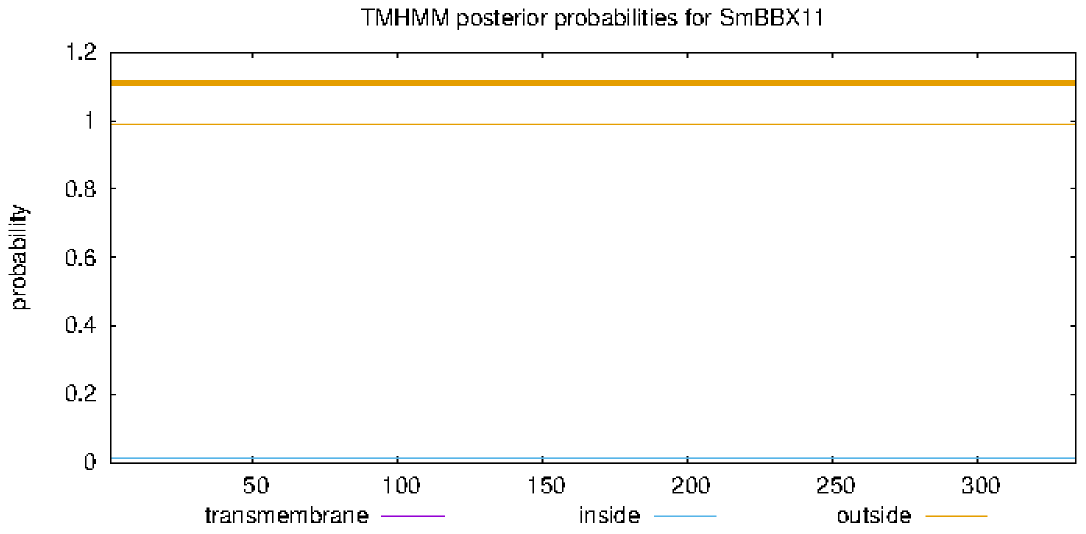
TMHMM posterior probabilities for SmBBX11
probability
1.2
1
0.8
0.6
0.4
0.2
0
50
100
150
200
250
300
transmembrane
inside
outside

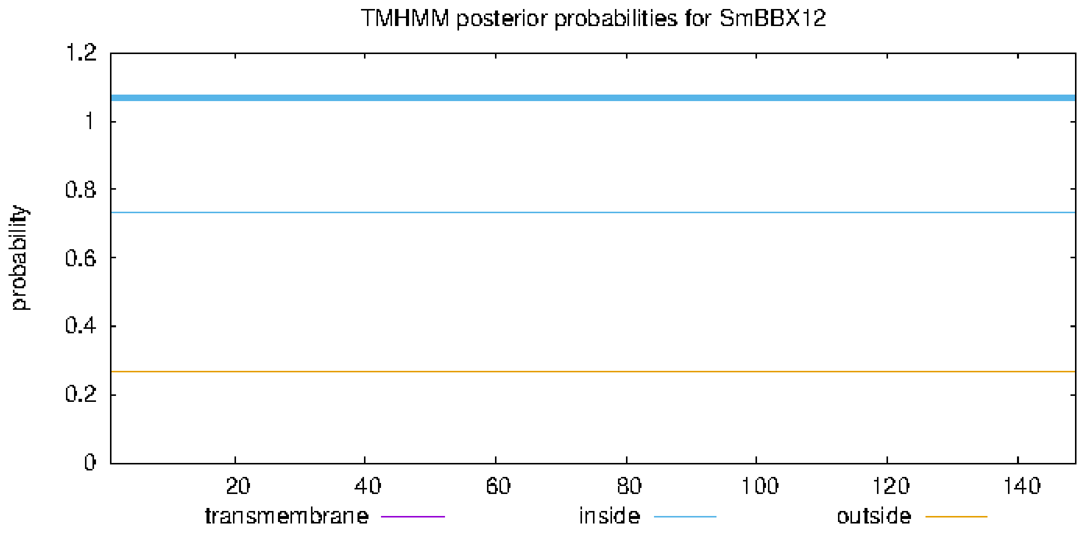
TMHMM posterior probabilities for SmBBX12
probability
1.2
1
0.8
0.6
0.4
0.2
0
20
40
60
80
100
120
140
transmembrane
inside
outside

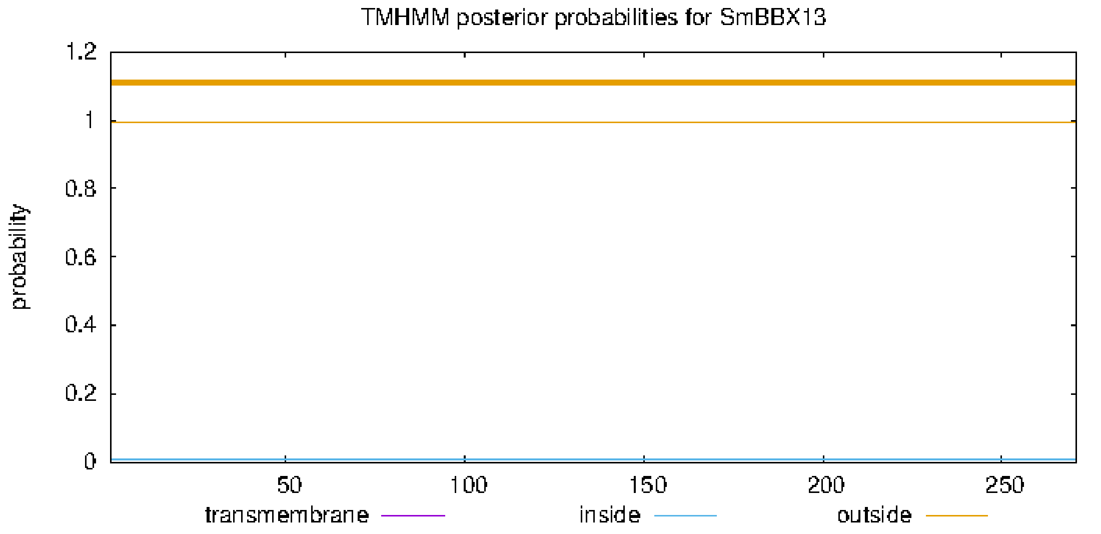
TMHMM posterior probabilities for SmBBX13
probability
1.2
1
0.8
0.6
0.4
0.2
0
50
100
150
200
250
transmembrane
inside
outside

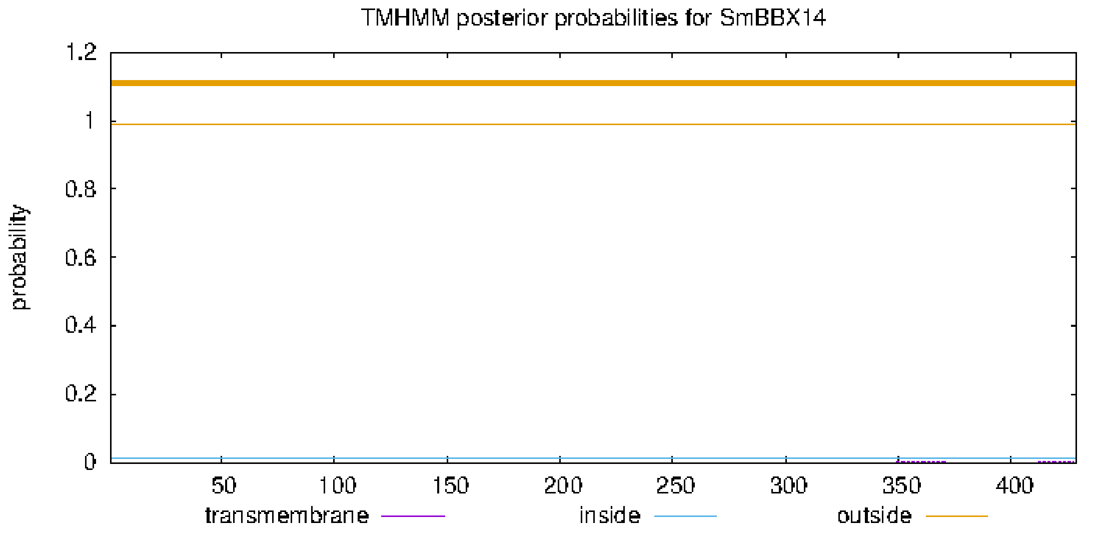
TMHMM posterior probabilities for SmBBX14
probability
1.2
1
0.8
0.6
0.4
0.2
0
50
100
150
200
250
300
350
400
transmembrane
inside
outside

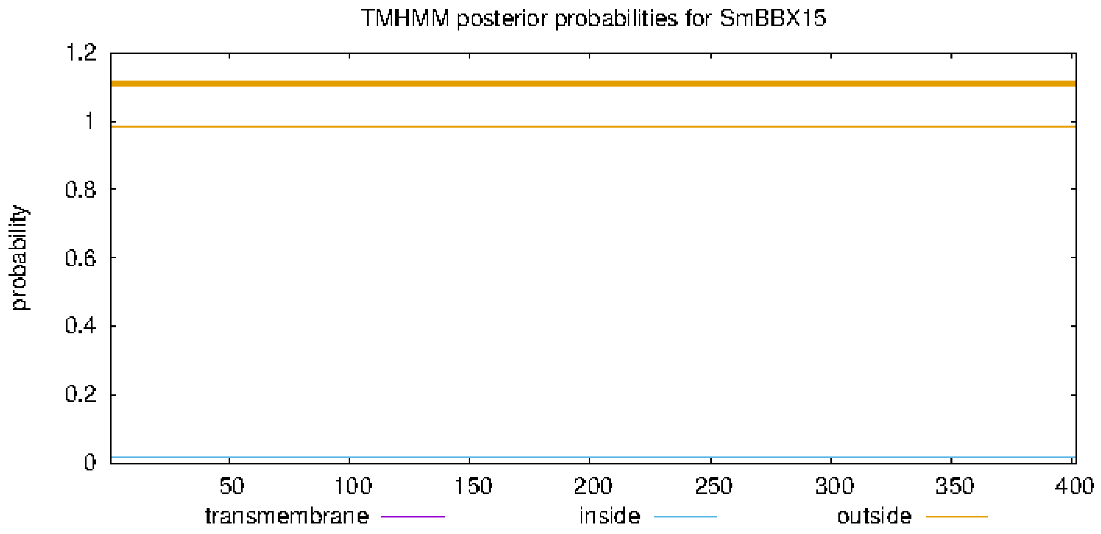
TMHMM posterior probabilities for SmBBX15
probability
1.2
1
0.8
0.6
0.4
0.2
0
50
100
150
200
250
300
350
400
transmembrane
inside
outside

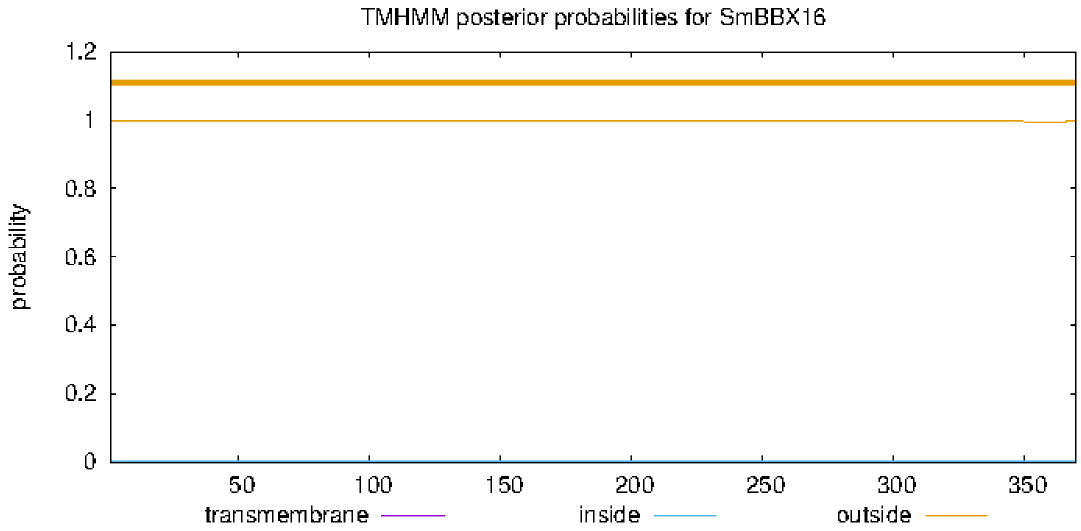
TMHMM posterior probabilities for SmBBX16
probability
1.2
1
0.8
0.6
0.4
0.2
0
50
100
150
200
250
300
350
transmembrane
inside
outside

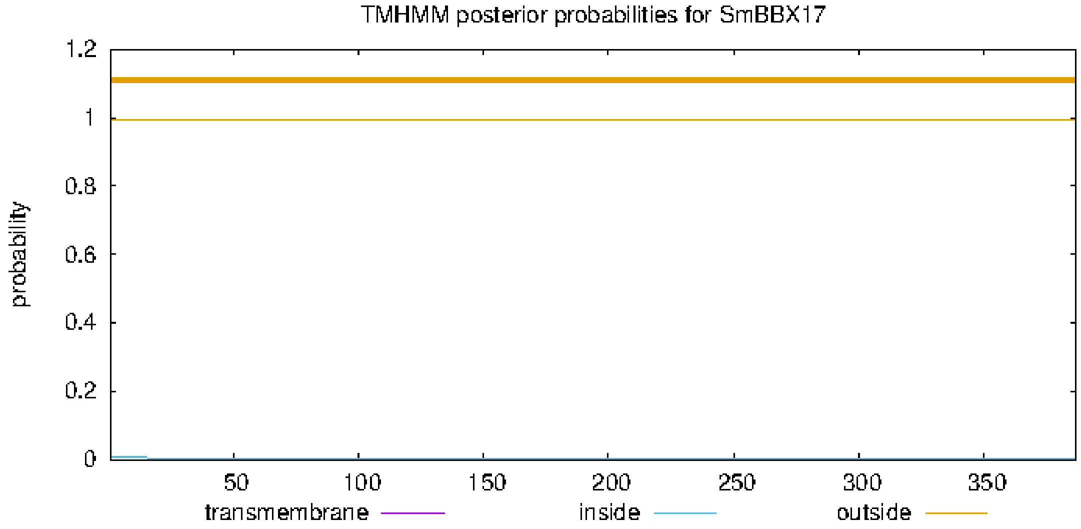
TMHMM posterior probabilities for SmBBX17
probability
1.2
1
0.8
0.6
0.4
0.2
0
50
100
150
200
250
300
350
transmembrane
inside
outside

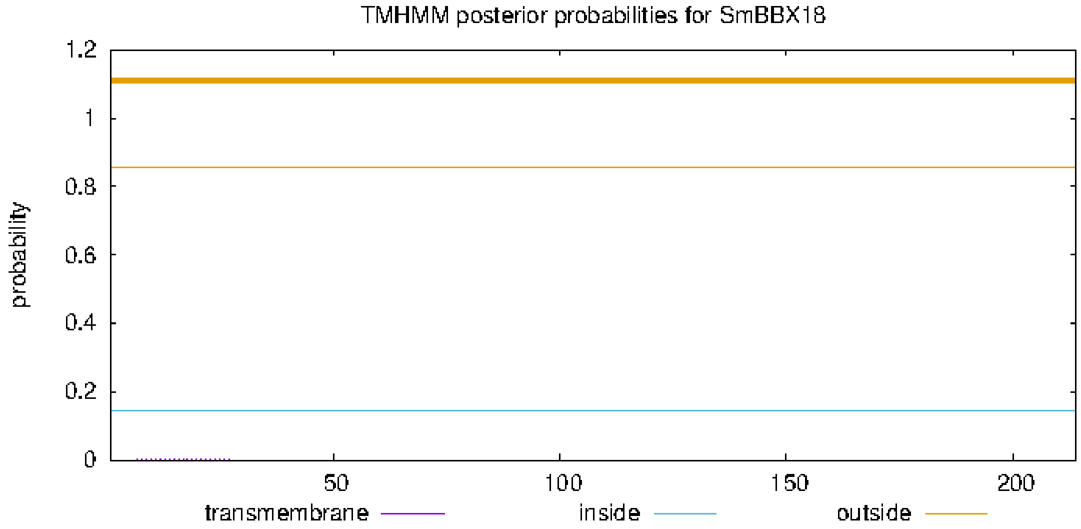
TMHMM posterior probabilities for SmBBX18
probability
1.2
1
0.8
0.6
0.4
0.2
0
50
100
150
200
transmembrane
inside
outside

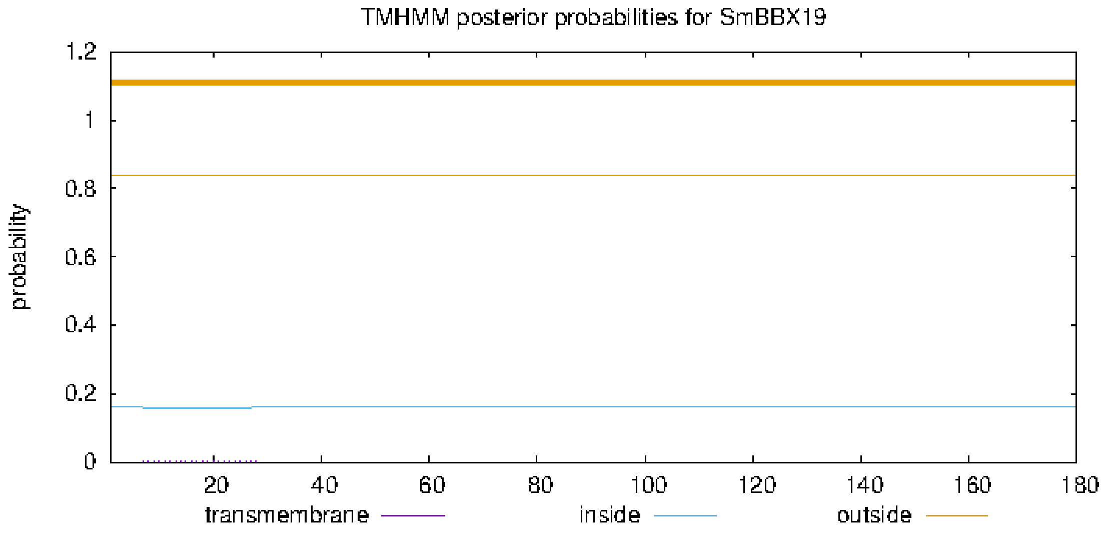
TMHMM posterior probabilities for SmBBX19
probability
1.2
1
0.8
0.6
0.4
0.2
0
20
40
60
80
100
120
140
160
180
transmembrane
inside
outside

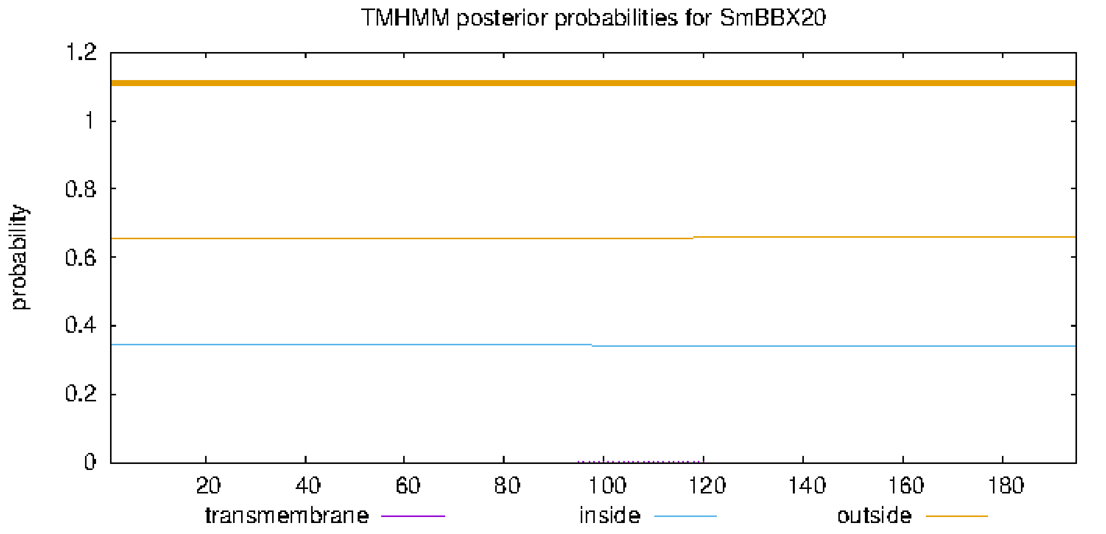
TMHMM posterior probabilities for SmBBX20
probability
1.2
1
0.8
0.6
0.4
0.2
0
20
40
60
80
100
120
140
160
180
transmembrane
inside
outside

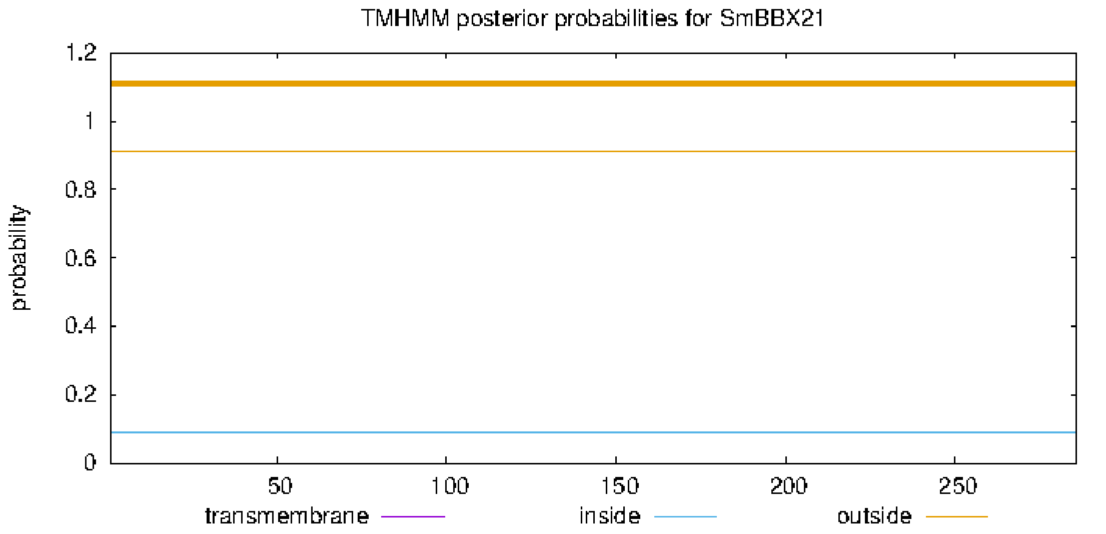
TMHMM posterior probabilities for SmBBX21
probability
1.2
1
0.8
0.6
0.4
0.2
0
50
100
150
200
250
transmembrane
inside
outside

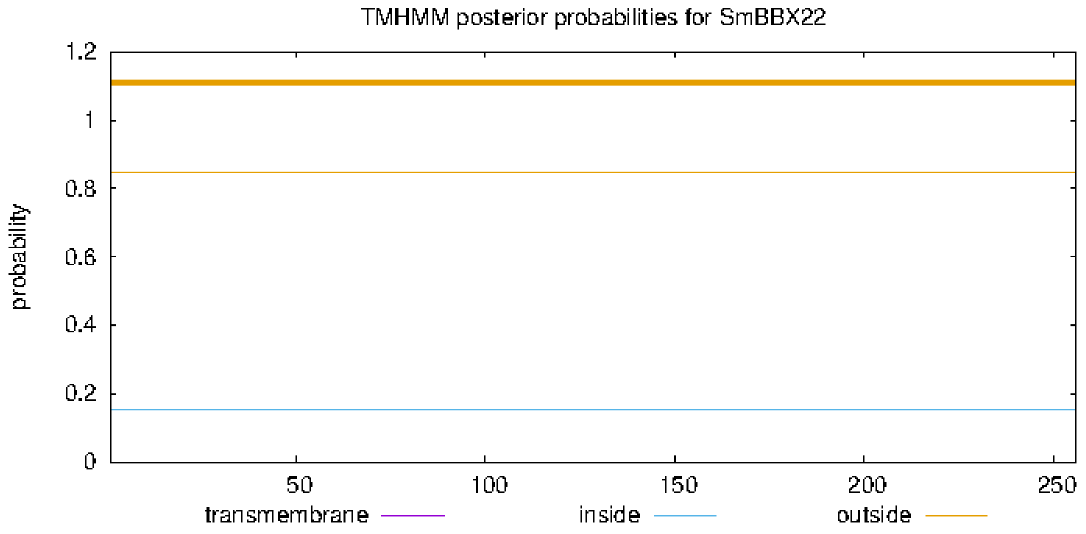
TMHMM posterior probabilities for SmBBX22
probability
1.2
1
0.8
0.6
0.4
0.2
0
50
100
150
200
250
transmembrane
inside
outside

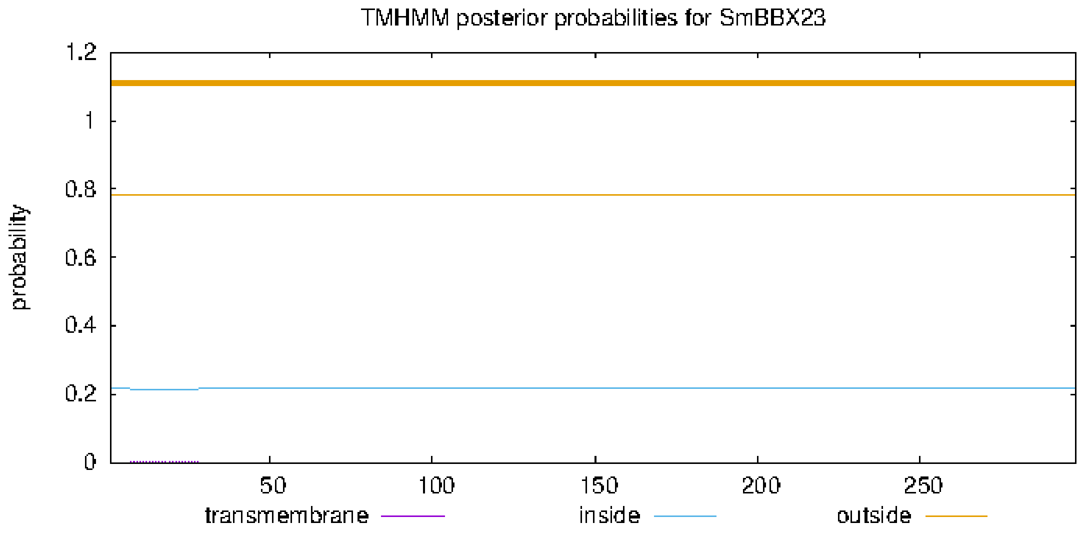
TMHMM posterior probabilities for SmBBX23
probability
1.2
1
0.8
0.6
0.4
0.2
0
50
100
150
200
250
transmembrane
inside
outside

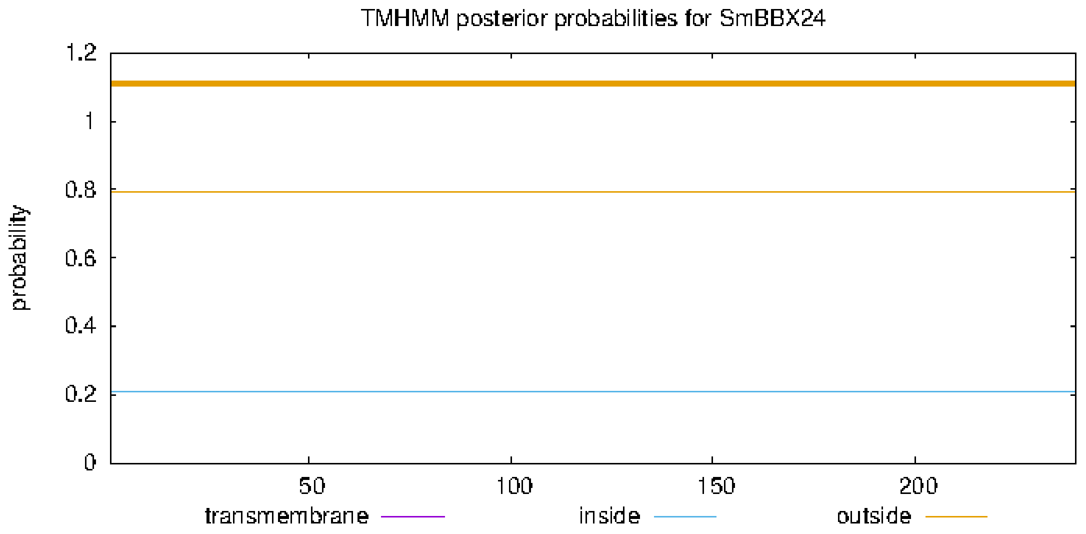
TMHMM posterior probabilities for SmBBX24
probability
1.2
1
0.8
0.6
0.4
0.2
0
50
100
150
200
transmembrane
inside
outside

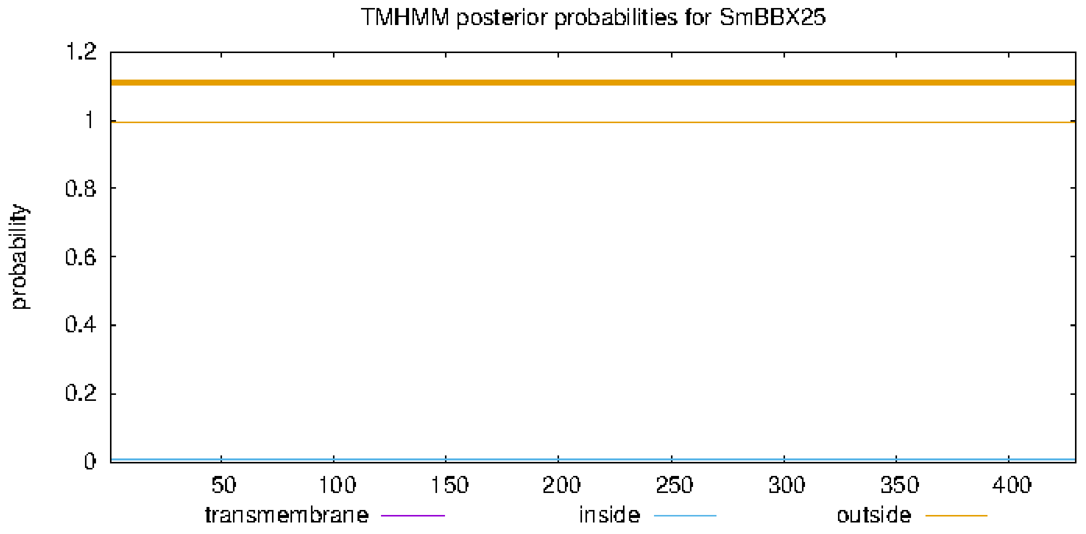
TMHMM posterior probabilities for SmBBX25
probability
1.2
1
0.8
0.6
0.4
0.2
0
50
100
150
200
250
300
350
400
transmembrane
inside
outside

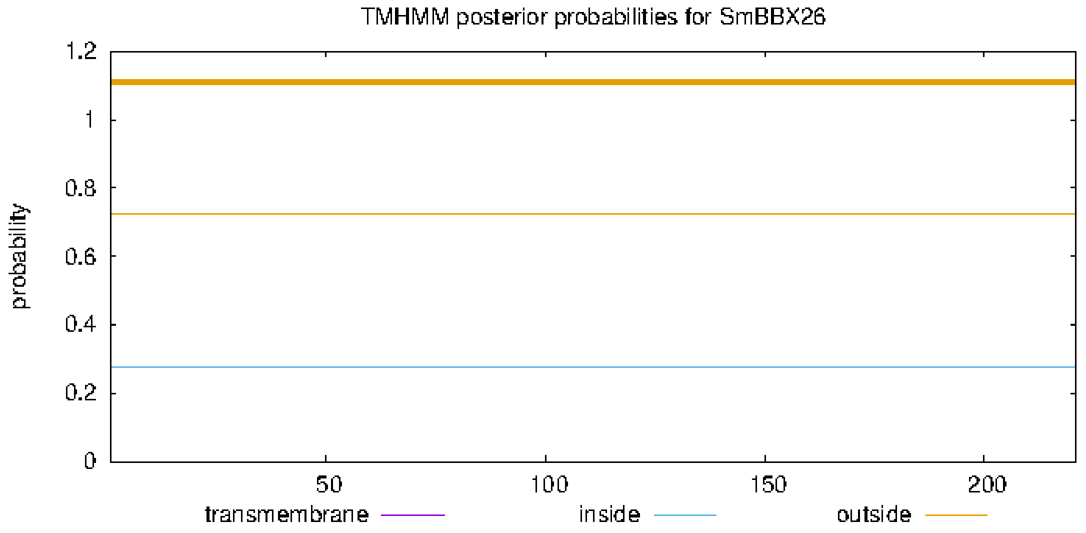
TMHMM posterior probabilities for SmBBX26
probability
1.2
1
0.8
0.6
0.4
0.2
0
50
100
150
200
transmembrane
inside
outside

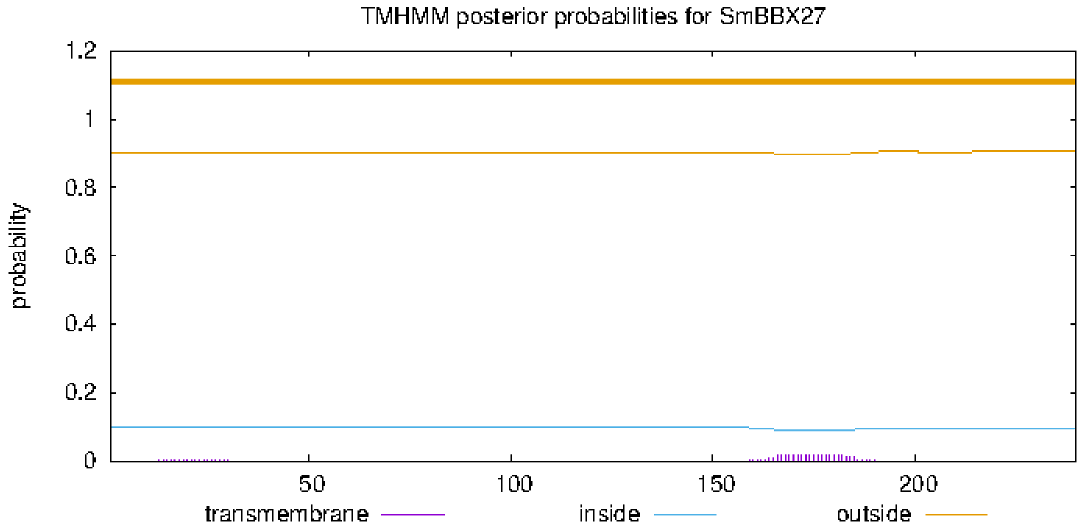
TMHMM posterior probabilities for SmBBX27
probability
1.2
1
0.8
0.6
0.4
0.2
0
50
100
150
200
transmembrane
inside
outside
